# Artificial intelligence for art investigation: Meeting the challenge of separating x-ray images of the *Ghent Altarpiece*

**DOI:** 10.1126/sciadv.aaw7416

**Published:** 2019-08-30

**Authors:** Z. Sabetsarvestani, B. Sober, C. Higgitt, I. Daubechies, M. R. D. Rodrigues

**Affiliations:** 1 Department of Electronic and Electrical Engineering, University College London, London, UK.; 2 Department of Mathematics and Rhodes Information Initiative, Duke University, Durham, NC, USA.; 3 Scientific Department, National Gallery, London, UK.; 4 Department of Electrical and Computer Engineering, Duke University, Durham, NC, USA.; 5 Alan Turing Institute, British Library, 96 Euston Road, London NW1 2DB, UK.

## Abstract

X-ray images of polyptych wings, or other artworks painted on both sides of their support, contain in one image content from both paintings, making them difficult for experts to “read.” To improve the utility of these x-ray images in studying these artworks, it is desirable to separate the content into two images, each pertaining to only one side. This is a difficult task for which previous approaches have been only partially successful. Deep neural network algorithms have recently achieved remarkable progress in a wide range of image analysis and other challenging tasks. We, therefore, propose a new self-supervised approach to this x-ray separation, leveraging an available convolutional neural network architecture; results obtained for details from the *Adam* and *Eve* panels of the *Ghent Altarpiece* spectacularly improve on previous attempts.

## Teaser

Artificial intelligence aids in the separation of x-ray images of two-sided paintings.

## INTRODUCTION

In the art investigation domain, increasing use of extremely high-resolution digital imaging techniques is being made in parallel with the widespread adoption of a range of recent imaging and analytical modalities not previously applied in the field (e.g., hyperspectral imaging, macro x-ray fluorescence scanning, and novel forms of imaging x-ray radiography) (*1*–*3*). These techniques mean that there is a wealth of digital data available within the sector, offering huge scope to provide new insights but also presenting new computational challenges to the domain (*4*).

In the past decades, various other disciplines, experiencing similar data growth, have benefited greatly from recent breakthroughs in artificial intelligence. The availability of cutting-edge machine learning algorithms, as well as the enhanced computation power and frameworks necessary to deal with massive datasets, have yielded outstanding results in computer vision, speech recognition, speech translation, natural language processing, and more (*5*).

It is therefore natural to develop similar techniques to address challenging tasks arising in art investigation (*6*), including material identification within different strata of paintings (*4*, *7*, *8*), analysis of brush stroke style (*9*, *10*), automatic canvas thread counting (*11*), digital inpainting of cracks (*12*, *13*), and visualization of concealed designs and underdrawings (*14*–*16*).

**Fig. 1:**
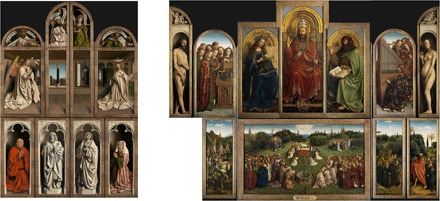
The *Ghent Altarpiece*: closed (left, shown after conservation) and open (right, shown before conservation; for these panels, conservation is ongoing and images after conservation are not available yet). The bottom left panel of the open left wing has been missing since its theft almost a century ago. [Images in this figure, and for details in further figures, are used with permission of the copyright holder, Saint-Bavo’s Cathedral (Art in Flanders; www.lukasweb.be). Photo credits: D. Provost (closed Ghent Altarpiece) and H. Maertens (open Ghent Altarpiece).]

This paper deals with a challenging image processing task arising in the context of the painting *The Adoration of the Mystic Lamb*, painted in 1432 by the brothers Hubert and Jan Van Eyck and more commonly known as the *Ghent Altarpiece*, shown in Fig. 1. This piece is one of the most admired and influential paintings in the history of art, showcasing the Van Eyck brothers’ unrivaled mastery of the oil painting technique (*17*, *18*). Over the centuries, the monumental polyptych (350 cm by 470 cm when open) has been prized for its stunning rendering of different materials and textures and its complex iconography. Originally, the polyptych consisted of four central panels, and then two wings each consisting of four panels painted on both sides so that entirely different sets of images and iconography could be seen depending on whether or not the wings were open (for example on Feast days). This paper focuses on two of the double-sided panels, depicting *Adam* and *Eve* on the interiors (and with the *Annunciation* and interior scenes on the outside).

Since 2012, this world-famous masterpiece has been undergoing a painstaking conservation and restoration campaign being undertaken by the Belgian Royal Institute for Cultural Heritage (KIK-IRPA). This treatment is being supported by an extensive research project using a diverse range of imaging and analytical techniques to inform and fully document the treatment, as well as provide new insights into the materials and techniques of the Van Eyck brothers and support art historical research. This ongoing project and most of the reports and resources it is generating, including high-resolution images of each panel, acquired using a range of different modalities (high-resolution visible images, high-resolution infrared photographs, infrared reflectographs, and x-radiographs), are fully accessible via the Closer to Van Eyck website (http://closertovaneyck.kikirpa.be/ghentaltarpiece/#home).

Of particular relevance, x-radiographs (x-ray images) are a valuable tool during the examination and restoration of paintings, as they can help establish the condition of a painting (e.g., whether there are losses and damages that may not be apparent at the surface, perhaps because of obscuring varnish, overpainted layers, structural issues, or cracks in the paint) and the status of different paint passages (e.g., help to identify retouchings or fills) (*19*). X-ray images can also be valuable in providing insights into artists’ technique and working methods and how they have used and built up different paint layers, or about the painting support (e.g., type of canvas or the construction of a canvas or panel). In some cases, it may also be possible to get some idea of the materials used (e.g., distinguish pigments on the basis of elements of a high atomic number like lead from pigments such as ochres or lake pigments, which contain elements of a low atomic number).

**Fig. 2:**
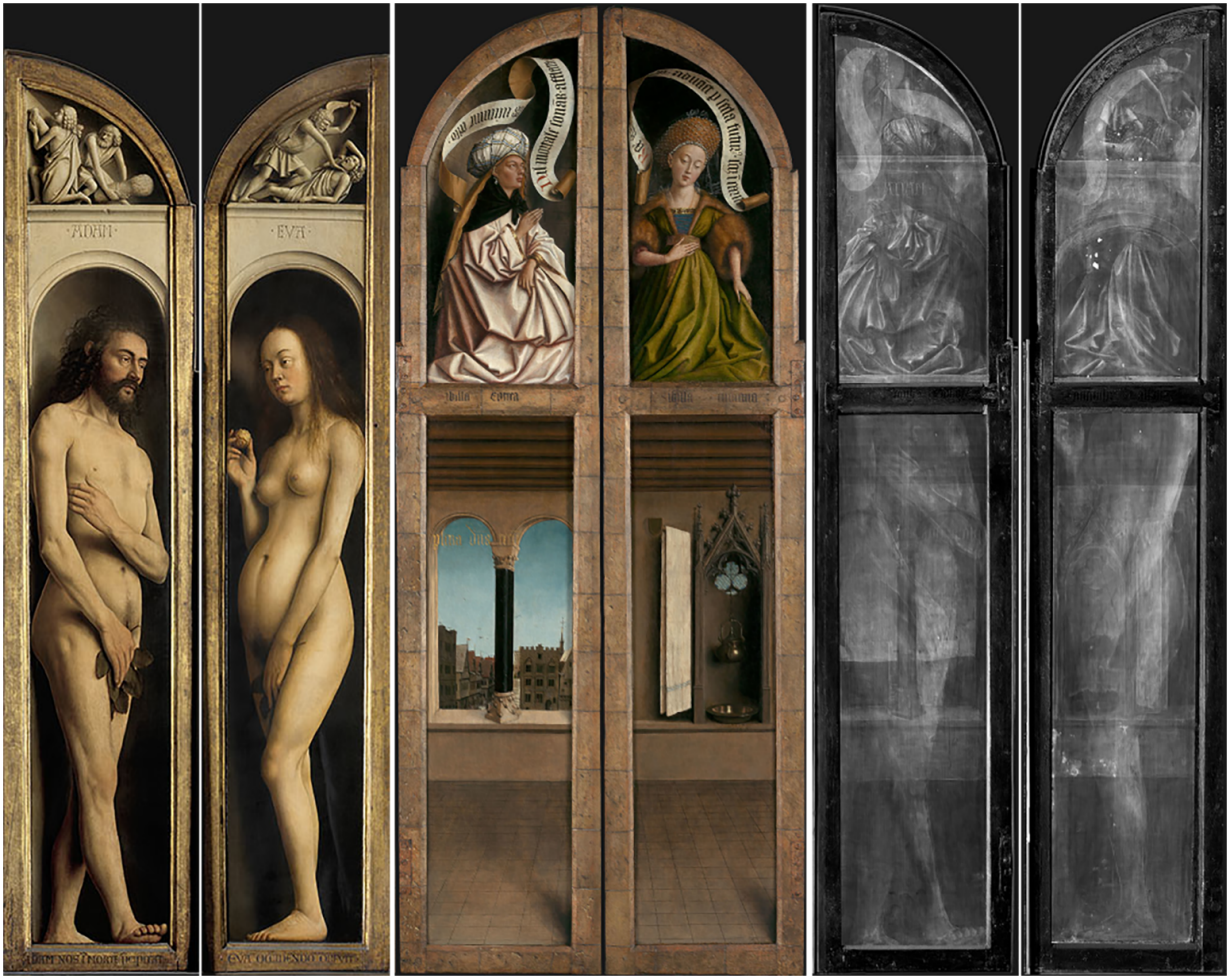
The two double-sided panels from the *Ghent Altarpiece*. Interior view of shutters (left, before conservation), exterior view of shutters (center, after conservation), and corresponding x-ray images for each panel (right, acquired before conservation), which include the combined contributions from both sides of each panel. Note that because x-ray imaging captures both sides by penetrating through the panel, whereas the panel has to be turned over to photograph its backside in visible light, the x-ray images superpose the mirror image of one side and the nonmirrored image of the other side. [X-ray image in this figure, and for details in further figures, are used with permission of the copyright holder, Saint-Bavo’s Cathedral (Art in Flanders; www.lukasweb.be). Photo credit: KIK-IRPA.]

However, interpreting x-ray images can be problematic. The attenuation of x-rays (and thus the brightness of the resulting region) depends not only on the atomic number of the material but also on its physical thickness. A further challenge is the fact that x-ray images are two-dimensional (2D) representations of 3D objects. While paintings are generally quite thin and flat, features at the front, back, or even within the painting will all appear in the radiograph. Thus, for example, the structure of the support (such as canvas weave, wood grain, or fixings used) will be visible as will cradles or stretcher bars (*20*, *21*). If the support is painted on both sides or if the design has been altered by the artist or a support has been reused, all of the images (or stages of development of an image) are visibly overlaid or “blended” together. In our case, the x-ray images of the *Adam* and *Eve* double-sided panels present a mixture of information from each side of the panels, impairing the ability of conservators to “read” them (see Fig. 2).

The challenge set forth in this paper is the separation of the mixed x-ray images from the double-sided panels into separate x-ray images of corresponding (imagined) “one-sided” paintings. Source separation of mixed signals has received much attention in the literature. Various algorithms concerning different scenarios associated with different levels of supervision have been proposed to solve this problem. These include unsupervised, semisupervised, and fully supervised approaches. The unsupervised (blind) source separation algorithms tackle the problem by adding different assumptions on the sources [e.g., non-Gaussianity (*22*), sparsity (*23*–*25*), and low rank (*26*)]. Semisupervised source separation frameworks, on the other hand, assume that we have access to a training set containing samples from the distribution of unmixed source signals (*27*, *28*). This prior information is exploited in the precomputation of dictionaries that represent the signals. Last, in a fully supervised source setting, it is assumed that we have access to a training set comprising both the mixture and the individual signals (possibly for different paintings by the same artist in a similar style), allowing the algorithm to learn a mapping from the mixture to the source signals (*29*). Here, we deal with yet another approach: source separation with side information. That is, we assume that we have some prior knowledge (not necessarily accurate) regarding the individual mixed sources; here, it is in the form of other images correlated with the mixed ones.

To address this source separation problem, we propose a convolutional neural network (CNN)–based self-supervised framework. This scheme posits access to a collection of signals, which are correlated with the source signals, as well as the mixed signal. In our case, to separate the mixed x-ray image into reconstructed x-ray images of each side, we train a deep neural network, leveraging the availability of the following: (i) the visible RGB image associated with the front of the panel, as well as (ii) that associated with the rear of the panel and (iii) the mixed x-ray image. Unlike other studies that train neural networks on large annotated datasets, and then use the network to solve some specific task, here, labeled training data are not available, because of the nature of the problem at hand. Instead of having large sets of labeled data to learn from, we use high-resolution images (allowing for the creation of a large number of input patches) and train the network based on implicit labeling; i.e., the mixed x-ray image. Explicitly, we fit a CNN model, which takes the standard visual imagery (RGB images) as input and generates two separated x-ray images as output. The learning process is done through minimizing the differences between (i) the sum of the reconstructed x-ray images and (ii) the original mixed x-ray image; hence, we call it self-supervised (for more details, please see Materials and Methods).

A number of approaches had already been explored in recent years to attempt to separate mixed x-ray images, relying on RGB images associated with double-sided paintings (*30*, *31*). These approaches—taking advantage of sparse signal and image processing techniques—had some partial success, with the main features from the front and rear sides appearing on the respective separated x-ray images. However, in those earlier results, both proposed reconstructed x-ray images continued to contain elements that belong to the other side (a comparison between the proposed approach to former ones is presented in Results below). It therefore appears that state-of-the-art approaches in signal and image processing to date are unable to satisfactorily tackle this x-ray image separation task.

## RESULTS

Our new self-supervised approach (see Materials and Methods) has been applied to two independent test image sets; explicitly, details from the *Adam* and *Eve* panels presented in Fig. 2. As will be elaborated further below, our final procedure comprises the training of two neural networks for each test case. The final results produced by this approach appear to present a near-perfect separation of the mixed x-ray images in both cases; they are obtained in several stages, as explained below.

**Fig. 3:**
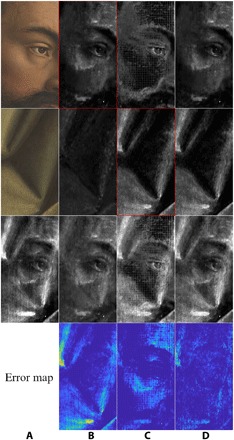
Results of the proposed algorithm applied to a detail from the *Adam* panel. Column (**A**) input data; (**B** to **D**) results from first (B), second (C), and combined (D) approach—the latter takes the best of both first and second approaches. Top row: Interior (*Adam*) side RGB input (before conservation) and various reconstructed x-ray images. Second row: Exterior (drapery) side RGB input (image mirrored for easier comparison with x-ray images) and the reconstructed x-ray images. Third row: Original mixed x-ray input image (left) and mixtures of the reconstructed x-ray images in rows 1 and 2. Bottom row: Visualization of the error map for each approach.

**Fig. 4:**
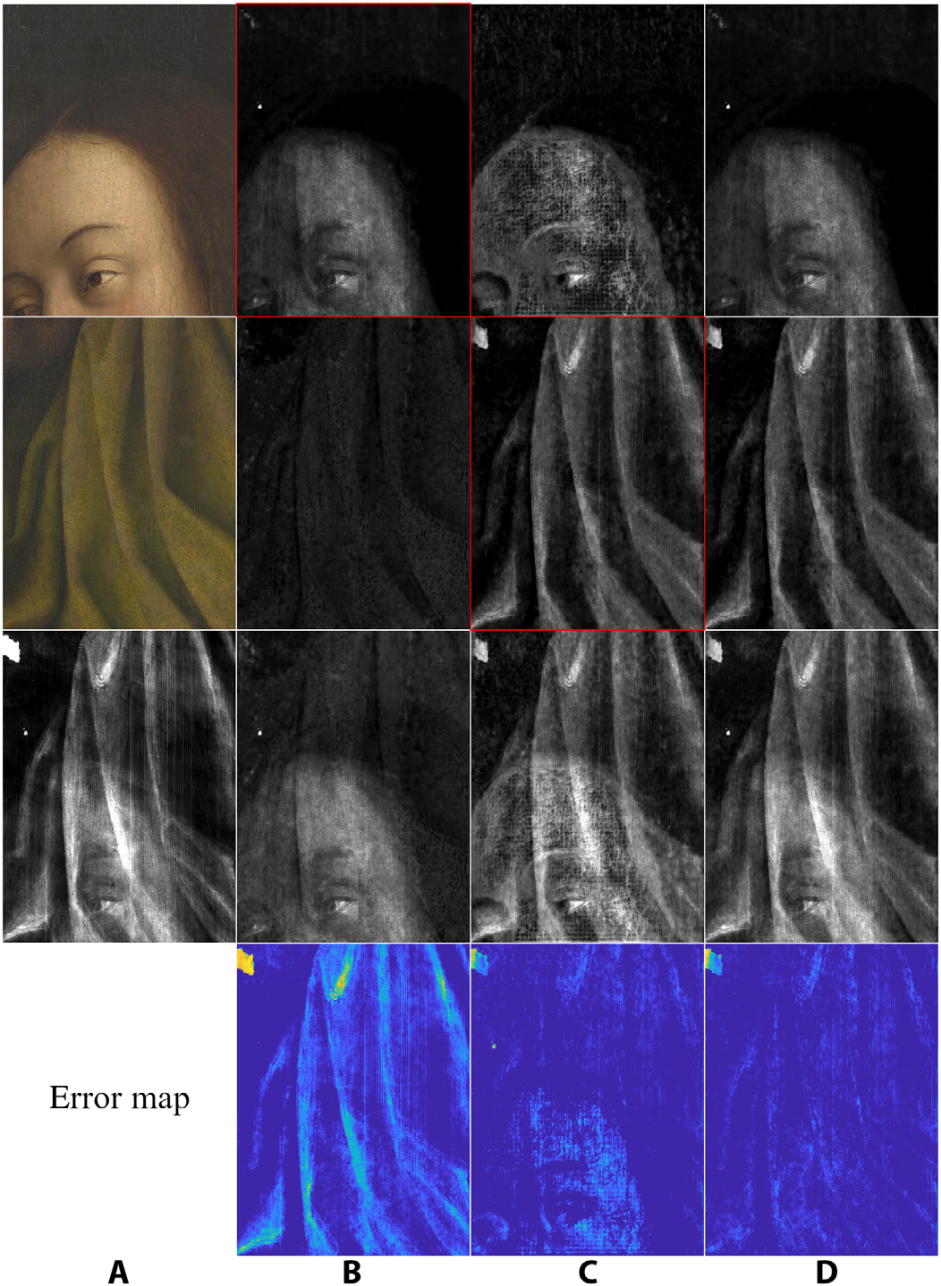
Results of the proposed algorithm applied to a detail from the *Eve* panel. Column (**A**) input data; (**B** to **D**) results from first (B), second (C), and combined (D) approach—the latter takes the best of both first and second approaches. Top row: Interior (*Eve*) (before conservation) and various reconstructed x-ray images. Second row down: exterior (drapery) side RGB input (image mirrored for easier comparison with the x-ray images) and the reconstructed x-ray images. Third row down: Original mixed x-ray input image (left) and mixtures of the reconstructed x-ray images in rows 1 and 2. Bottom row: Visualization of the error map for each approach.

Initially, we attempted to learn a conditioned mapping *f_x_*(⋅) : *y_k_* → *x_k_* from the RGB images *y*_1_ and *y*_2_ to separated x-ray images *x*_1_ and *x*_2_, given the mixed x-ray *x* (see below the “First approach” subsection in Materials and Methods). This approach already yielded better results than other state-of-the-art methods designed to perform such separations (see column B in Figs. 3 and 4, showing the results from the first approach for the *Adam* and *Eve* panels; in both cases, the reconstructed x-ray for the interior side, framed in red, is the better reconstruction of the two). The mean squared error (MSE; per pixel) of this first approach is 0.0094 for the *Adam* panel and 0.0053 for the *Eve* panel (with grayscale values ranging from 0 to 1); this is the average mean square deviation, over the extent of the whole detail image, of the pixel value for the mixture *x*_1_ + *x*_2_ of the two reconstructed x-ray images computed by the algorithm, compared with the pixel value of the input (mixed) x-ray image *x*.

However, we noticed that the reconstruction of the x-ray image of the side corresponding to the first input *y*_1_ (the interior side, with the eyes of the *Adam* and *Eve* visible images) is much more faithful than that of the side corresponding to the other input *y*_2_ (the exterior side, figuring portions of drapery); see column B in Figs. 3 and 4. Upon switching the order of the inputs *y*_1_ and *y*_2_, we obtained a better reconstruction of the x-ray image of the other side (see column C in Figs. 3 and 4, which show the results from the second approach where the input order is swapped and where the reconstructed x-ray for the exterior sides of the panels, framed in red, is the better one, for each of the two examples). This indicated, to our surprise, that our method was far from being indifferent to the ordering of the input data, although we would expect such invariance for symmetric cost functions such as the one we optimize for; see Eq. 2 in Materials and Methods. With this reversed order of inputs, the MSE was 0.0145 for the *Adam* panel and 0.0126 for the *Eve* panel. (It is noticeable that upon swapping the input, the error jumps. This may be explained by the fact that the x-ray data are more correlated with the faces’ sides than that of the textiles’, maybe due to a possibly more pronounced presence of x-ray–absorbing ingredients in the pigments used on the faces’ side.)

As will be explained at greater length in Materials and Methods, we thus propose combining the best of the two available x-ray image reconstructions (one for each order of inputs) to build a combined x-ray reconstruction; this combined result provides the most accurate reconstruction of the mixed x-ray not only on the basis of visual inspection (see column D in Figs. 3 and 4) but also on the basis of the MSE, yielding 0.0016 for the *Adam* panel and 0.0020 for the *Eve* panel. As can be expected this phenomenon occurs even when we take the mean absolute error as our measure (see Materials and Methods for the exact values).

**Fig. 5:**
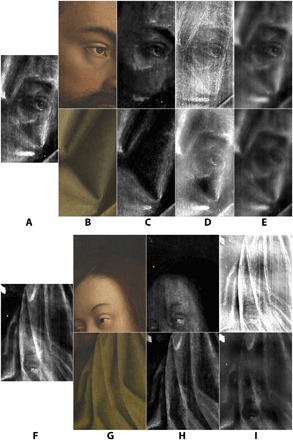
Comparison between the results generated with the proposed new algorithm and the preceding state-of-the-art results for a detail of the *Adam* panel. Top: (**A**) the mixed x-ray; (**B**) the RGB images from each side of the panel (before conservation) corresponding to the x-ray detail (i.e., the algorithm inputs); (**C**) reconstructed x-ray images produced by the proposed algorithm; (**D**) reconstructed x-ray images produced in (*25*); and (**E**) reconstructed x-ray images produced by coupled dictionary learning (*30*). All of the grayscale images presented here have gone through histogram stretching to have a common ground for the comparison. *Eve* panel (bottom): (**F**) the mixed x-ray; (**G**) the RGB images from each side of the panel (before conservation) corresponding to the x-ray detail (i.e., the algorithm inputs); (**H**) reconstructed x-ray images produced by the proposed algorithm; and (**I**) reconstructed x-ray images produced in (*30*). All of the grayscale images presented here have gone through histogram stretching to have a common ground for the comparison.

Visual evaluation of the results, achieved using our proposed approach, shows a spectacularly improved separation of the individual x-ray images, while the reconstructed mixture of x-ray images is nearly exact, as can be verified by checking the error maps in Figs. 3 and 4. In particular, the two separated images seem to contain elements pertaining to just one side of each panel (the very bright feature in the top left of the *Eve* panel is probably a fill with a very x-ray opaque material; interestingly the algorithm puts it on the textile side of the panel). As can be seen in Fig. 5, this was not the case with former cutting-edge methods, such as those presented in (*25*, *30*). Visual comparison with the earlier results shows clear potential of the usefulness of our present algorithm for art historians, whereas the former methodologies failed to yield a trustworthy separation. (Honesty compels us to add that MSE per pixel of our new, visually superior results is larger by more than an order of magnitude than that for the approach illustrated in column E of Fig. 5 (top part). In the present case, we feel that this is really one more illustration of the well-known shortcoming of MSE per pixel as a measure of quality of image reconstruction (*32*). In the earlier comparisons we made, using the MSE per pixel to compare *x*_1_ + *x*_2_ with *x* among the first, second, and combined approaches made a bit more sense because the images were more similar in nature; however, it was not as meaningful in comparing the approaches illustrated in column C against column D or E of Fig. 5 as the quality of separation is not being measured in this way.

## DISCUSSION

The results reported above present a big step forward in the ability to unmix x-radiographs of two-sided paintings into two separate x-ray images, each corresponding to just one side of the painting. It is clear from visual inspection that the separations, reported here, are exceedingly better than former state-of-the-art approaches.

As explained in more detail in Materials and Methods, our method relies on choosing “the best of all possible worlds” (borrowing Voltaire’s words), by taking the first output of two separate runs, differing only in the ordering of the inputs. We have no good explanation why “cutting and pasting together” pieces of results obtained from two different optimization processes would lead to a more optimal result for the same cost function (see Eq. 2 for the definition of the cost function). One possible surmise, which may explain such behavior, is that the asymmetry is a result of the way the TensorFlow package’s optimization [(*33*); this package was used in all of our experiments] is being implemented internally. Thus, each of the optimization processes might get stuck in some local minimum, nicely matching the first x-ray image.

It is noteworthy that the MSE measure, reported throughout the paper, does not correlate in full with the quality of the reconstructions (and even yields better MSE for formerly used methods). In other words, this way of measuring the error does not take into account how well the mixed signal is being separated into two independent signals. Furthermore, the fact that per-pixel loss function does not capture well the perceptual loss is a long-standing issue in image processing [e.g., (*32*)]. In our case, the limitations of the MSE are even sharper, as by taking one of the reconstructed images to be exactly the mixed x-radiograph and the other to be just a black image, the MSE would yield 0. Although we have reservations regarding the reliance on MSE in image analysis, it is still the most popular and convenient way to measure errors, and hence, we used it in our optimization.

Be that as it may, the empirical results in our application, shown in two independent experiments (i.e., two different panels of the *Ghent Altarpiece*) in Fig. 5, do not leave room for doubt: This approach works and seems to give remarkable results. Such unexpected outcome calls for further investigation, possibly into the nature itself of the neural network application—an appealing prospect in its own right, since the singular effectiveness of deep neural networks is not well understood, and any peculiar behavior, in any of its successful implementations, can possibly be used to unlock new insights. Just as when an experimental physicist conducts an experiment yielding exceptional results not yet explained by theory, the reconstructions displayed above call for further exploration of the neural network’s design and the evolution of the various layers during the learning process.

Another important step in extending this research is for conservators, art historians, and heritage scientists to study the resulting reconstructed and separated x-ray images in detail, in conjunction with the other available technical data, to establish what new insights they can provide in terms of understanding the condition and creation of the paintings on the inner and outer sides of *Adam* and *Eve* panels. Such scrutiny will also be important in determining how well the separation algorithms have worked (in the sense of “assigning” the correct features to the correct image) and in characterizing the artifacts or blind spots inherent to the new method; such characterization will help guide further users.

In the *Ghent Altarpiece*, as in other panels from polyptych wings that have not been separated, both sides of the panels are fully accessible visually. However, we also intend to extend the approach developed for the *Adam* and *Eve* panels and see if it can help separate other examples of mixed x-ray images where one (or possibly more) of the contributing images is not visible. Examples where superimposed images or features may contribute to mixed x-ray images include reused supports [e.g., reused canvases in two works from the British National Gallery: Rembrandt’s *Portrait of Frederick Rihel on Horseback*, NG6300 (*34*) and Karel du Jardin’s *Portrait of a Young Man*, NG1680 (*35*)] or paintings with obvious pentimenti where the artist has altered a composition [e.g., the figures in Titian’s *The Death of Actaeon*, NG6420 (*36*)].

In addition, in recent years, there is a rapid growth in the availability and utilization of additional imaging modalities (e.g., macro x-ray fluorescence scanning, hyperspectral imaging, and spectroscopy) in the context of cultural heritage science. These imaging methods provide us with various ways of quantifying the properties of materials present in the artifact. Thus, certain modalities may be helpful in providing additional information about the surface or inaccessible concealed images, for example. Accordingly, cases of superimposed images where one image is completely visually inaccessible, but a range of multimodal images are available, could potentially benefit from the development of similar approaches to the one presented in this paper [e.g., in Francisco de Goya’s *Doña Isabel de Porcel*, NG1473 (*37*), Vincent van Gogh’s *Patch of Grass* (*14*), and Edgar Degas’s *Portrait of a Woman* (*15*)].

All of the abovementioned prospects suggest the need for a new research effort in the area of artificial intelligence for art investigation, an area presenting many unique challenges. For example, the data being collected are of immense complexity involving the use of a number of different multidimensional modalities (such as hyperspectral imaging, x-ray fluorescence and x-ray diffraction scanning, infrared reflectography or spectroscopy, Raman spectroscopy, and other methods). It is becoming clear that the utilization of such complex imaging and analytical techniques is likely to intensify in the coming years with the increasing availability, portability, and usability of instrumentation. Therefore, the development of new algorithms capable of ingesting such complex datasets will not only have far-reaching implications for art investigation but can open entirely new vistas both in computer and heritage science.

## MATERIALS AND METHODS

### Data and definitions

In the experiments performed, we attempted to separate mixed x-ray images of two details taken from two-sided panels of *Adam* and *Eve* of the *Ghent Altarpiece* (see Fig. 2). We denote henceforth the details corresponding to *Adam* and *Eve* as details 1 and 2, respectively. The resolution of both the x-ray and RGB images of details 1 and 2 were 604 × 331 and 852 × 630 pixels (see column A in Figs. 3 and 4), respectively. As a preprocessing step, we performed histogram stretching (normalization) for the mixed x-ray images (this same procedure was applied to all of the results presented in the article so that we would have a common ground for evaluation). Explicitly, we removed the upper and lower 1% grayscale values to avoid outliers, and then stretched the dynamic range between 0 and 255.

Let *S* denote a double-sided painting detail, and let *y*_1_ and *y*_2_ be two RGB images portraying the two sides of *S*. Let *x* denote the x-ray image of the panel, which encompasses information of the drawing from both sides of *S*. Because of the attenuation of x-rays as they pass through the support, *x* was a nonlinear combination of both sides of the panel. However, the effect was slight since the paint layer is rather thin, and the panel was almost transparent for the x-ray frequency used and can, therefore, be neglected by using a first-order approximation (amounting to restricting to the first term of the Taylor expansion). Accordingly, we model the observed x-ray *x* as the direct sum of two x-ray images *x*_1_ and *x*_2_

$$x=x_{1}+x_{2}$$ (1)

where *x*_1_ and *x*_2_ are the theoretical individual x-ray images corresponding to details 1 and 2. Our overarching goal was to recover the individual x-ray images *x*_1_ and *x*_2_ given the mixed x-ray image *x* and the individual RGB images *y*_1_ and *y*_2_.

### First approach: Self-supervised neural network

**Fig. 6:**
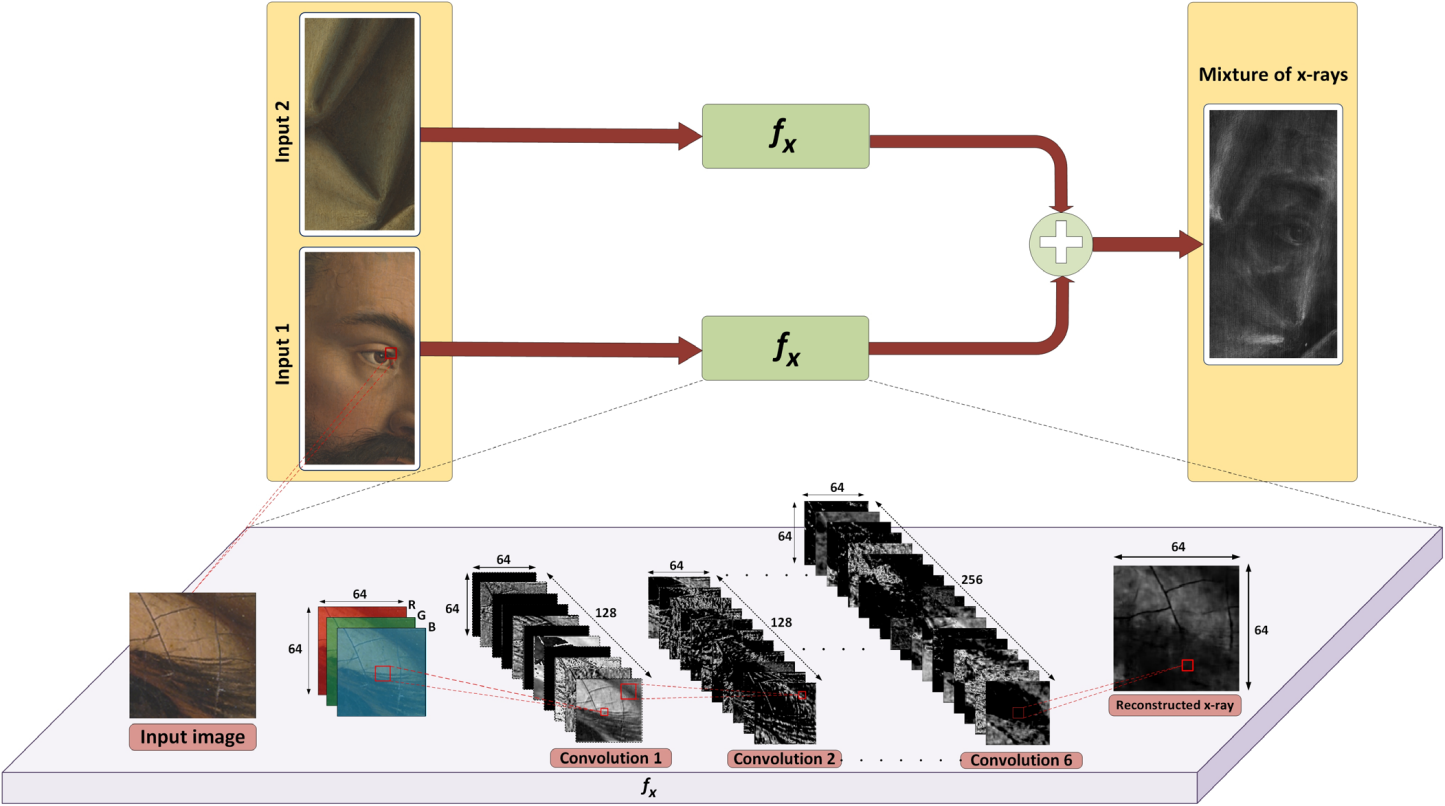
A diagram of the neural network architecture.

In our initial attempt, we designed a self-supervised neural network that learns how to convert (approximately) an RGB image onto an x-ray image. Figure 6 depicts a high-level abstraction of this proposed approach. Explicitly, our approach was based on the following principles:

1. The function *f_x_*( ⋅ ) : *y_k_* → *x_k_* maps the visual image associated with detail *k* onto the corresponding x-ray image.

2. The function *f_x_* is implemented using a CNN.

3. The function is being learned by minimizing

$$∥x-(f_{x}(y_{1})+f_{x}(y_{2})){∥}_{F}^{2}$$ (2)

so that conceptually, the mapping *f_x_*( ⋅ ) : *y_k_* → *x_k_* is converting an RGB image onto a corresponding x-ray in such a way that the linear superposition of the generated x-ray images corresponds to the available mixed x-ray.

4. The input corresponds to patches taken from *y*_1_ and *y*_2_, and the self-supervision is achieved through optimizing *f_x_* with respect to the counterpart patch from *x*.

The original images *y*_1_, *y*_2_, and *x* were taken as a collection of 64 × 64 patches with an overlap of 52 pixels resulting overall in roughly 966 and 3168 patch triplets for details 1 and 2, respectively. That is, the input data were organized as RGB *N* patches $(y_{1}^{j},y_{2}^{j})\in {\mathbb{R}}^{64\times 64\times 3}\times {\mathbb{R}}^{64\times 64\times 3}$ with the corresponding target patches *x^j^* ∈ ℝ^64 × 64 × 1^. We then constructed a seven-layer CNN along with batch normalization and rectified linear unit (ReLU) activation layers in between each of the convolution layers. The structure of the proposed network was inspired by the structure of pix2pix, which is an acceptable design for image-to-image translation using conditional adversarial network (*38*). Since its release, the pix2pix network model has attracted the attention of many internet users including artists (*39*). In our case, because of the lack of training data, we were unable to perform supervised adversarial training. Hence, we used only the “generator” network, and after experimenting with various structures, we observed that using only the encoder part of the generator provides the best reconstruction for x-ray images. Furthermore, our model deliberately overfitted the data as we were training and testing with the same dataset (i.e., a self-supervised learning). Therefore, we avoided using any sort of regularizer in the network structure.

For each of the seven convolutional layers (denoted by *l*_1_, *l*_2_, …, *l*_7_), we performed convolution with masks ${\left\{M_{k,i}\right\}}_{k=1}^{N_{i}}$, where the size of each mask was 5 × 5 × *N*_*i* − 1_. Accordingly, the output of each of these layers would be *N_i_* patches of size 64 × 64. We used *N*_0_ = 3, as the input layer comprises RGB color patches; for *i* = 1,2,3 we used *N_i_* = 128, and for *i* = 4,5,6 we used *N_i_* = 256; lastly, in the final layer providing the reconstructed x-ray, *N*_7_ = 1 to achieve a single 64 × 64 patch as the final outcome (see the network architecture on Fig. 6). Explicitly, given an input patch *p* ∈ ℝ^64 × 64 × 3^, the output of the layers is defined as

$$l_{i,k}=M_{1,k}*l_{i-1}+c_{i,k},\;\forall k=1,\ldots N_{i}$$ (3)

where *l_i_* ∈ ℝ^64 × 64 × *N*_*i* − 1_^ comes from stacking the *l*_*i*, *k*_ after batch normalization and activation, *l*_0_ = *p*, and *c*_*i*, *k*_ is a bias scalar valued parameter.

The learning process of the neural network aims at finding the most fitting entries of ${\left\{M_{k,i}\right\}}_{k=1}^{N_{i}}$, as well as *c*_*i*, *k*_. The optimization of these parameters, with respect to the cost function of Eq. 2, was done through random initialization and performing 300 iterations of stochastic gradient descent. A schematic drawing of the CNN architecture is shown in Fig. 6.

As a result of the network’s design, the resolution of the output images was the same as that of the input images. As can be seen in Figs. 3 and 4 (column B), the results yielded by this process gave a seemingly clean reconstruction of *x*_1_ and a substantially worse reconstruction of *x*_2_. However, even this result already improved upon other techniques designed to deal with the same problem (see Fig. 5). To check how faithful the reconstruction was to the mixed x-ray, we measured the MSE of the difference between the original mixed x-ray image and the summation of the two reconstructed separate x-ray images. The reconstruction MSE achieved by this approach was 0.0094 and 0.0053 (for grayscale values ranging between 0 and 1) when applied to details 1 and 2, respectively. The corresponding reconstruction mean absolute errors achieved by this approach were 0.0464 and 0.0297.

### Second approach: Reorder and combine

To test whether the order of the inputs mattered in the asymmetry of the quality of reconstruction of *x*_1_ and *x*_2_, noted above, we tried feeding a new CNN of the same structure with inputs in reversed order. Explicitly, instead of using the input data ${\left\{(y_{1}^{j},y_{2}^{j})\right\}}_{j=1}^{N}$, we used ${\left\{(y_{2}^{j},y_{1}^{j})\right\}}_{j=1}^{N}$. Since the cost function of Eq. 2 is symmetric, our expectation was that the results should be roughly the same. However, as can be seen in Figs. 3 and 4, the quality of the reconstructions of this run were in reverse order as well: The reconstruction of *x*_2_ was now far better than that of *x*_1_, and the MSE was now 0.0145 and 0.0126 when applied on details 1 and 2, respectively, which was roughly the same as in the first approach. The corresponding reconstruction mean absolute errors achieved by this approach were 0.0561 and 0.0495.

Seeing the two outcomes, we decided to mash them together into a single reconstruction and enjoy the benefits of both of them. That is, we wished to have a reconstruction comprising *x*_1_ of the first approach and *x*_2_ of the second attempt as our final result. More explicitly, adding another label to the outputs to indicate the order of inputs, so that $x_{i}^{\left[21\right]}$ indicates the output *i* when the inputs are ordered (*y*_2_, *y*_1_, and *x*), we posit our output, the pair $\left(x_{1}^{\left[12\right]},x_{2}^{\left[21\right]}\right)$. To our amazement, we found that the MSE of this combination yields 0.0016 and 0.0020 when applied on details 1 and 2, respectively (the mean absolute errors here were 0.0175 and 0.0171).

## References

[1] Alfeld M., Broekaert J. A. C., Mobile depth profiling and sub-surface imaging techniques for historical paintings—A review. Spectrochim. Acta Part B At. Spectrosc. 88, 211–230 (2013).

[2] Alfeld M., de Viguerie L., Recent developments in spectroscopic imaging techniques for historical paintings - a review. Spectrochim. Acta Part B At. Spectrosc. 136, 81–105 (2017).

[3] Huang X., Uffelman E., Cossairt O., Walton M., Katsaggelos A. K., Computational imaging for cultural heritage: Recent developments in spectral imaging, 3-D surface measurement, image relighting, and X-ray mapping. IEEE Signal Process. Mag. 33, 130–138 (2016).

[4] Delaney J. K., Conover D. M., Dooley K. A., Glinsman L., Janssens K., Loew M., Integrated x-ray fluorescence and diffuse visible-to-near-infrared reflectance scanner for standoff elemental and molecular spectroscopic imaging of paints and works on paper. Herit. Sci. 6, 31 (2018).

[5] LeCun Y., Bengio Y., Hinton G., Deep learning. Nature 521, 436–444 (2015). 26017442 10.1038/nature14539

[6] B. Cornelis, A. Dooms, I. Daubechies, P. Schelkens, Report on digital image processing for art historians, SAMPTA’09 (2009), pp. Special–session.

[7] Rohani N., Pouyet E., Walton M., Cossairt O., Katsaggelos A. K., Nonlinear unmixing of hyperspectral datasets for the study of painted works of art. Angew. Chem. 130, 11076–11080 (2018). 10.1002/anie.201805135 29940088

[8] Grabowski B., Masarczyk W., Głomb P., Mendys A., Automatic pigment identification from hyperspectral data. J. Cult. Herit. 31, 1–12 (2018).

[9] Johnson C. R., Hendriks E., Berezhnoy I., Brevdo E., Hughes S., Daubechies I., Li J., Postma E., Wang J. Z., Image processing for artist identification – computerized analysis of Vincent van Gogh’s painting brushstrokes. IEEE Signal Process. Mag. 25, 37–48 (2008).

[10] S. Jafarpour, G. Polatkan, E. Brevdo, S. Hughes, A. Brasoveanu, I. Daubechies, Stylistic analysis of paintings using wavelets and machine learning, in *17th European Signal Processing Conference* (IEEE, 2009), pp. 1220–1224.

[11] Johnson D. H., Johnson C. R. Jr., Erdmann R. G., Weave analysis of paintings on canvas from radiographs. Signal Processing 93, 527–540 (2013).

[12] Cornelis B., Ružić T., Gezels E., Dooms A., Pižurica A., Platiša L., Cornelis J., Martens M., De Mey M., Daubechies I., Crack detection and inpainting for virtual restoration of paintings: The case of the ghent altarpiece. Signal Processing 93, 605–619 (2013).

[13] T. Ružić, B. Cornelis, L. Platiša, A. Pižurica, A. Dooms, W. Philips, M. Martens, M. De Mey, I. Daubechies, International Conference on Advanced Concepts for Intelligent Vision Systems (Springer, 2011), pp. 417–428.

[14] Anitha A., Brasoveanu A., Duarte M., Hughes S., Daubechies I., Dik J., Janssens K., Alfeld M., Restoration of x-ray fluorescence images of hidden paintings. Signal Processing 93, 592–604 (2013).

[15] Thurrowgood D., Paterson D., De Jonge M. D., Kirkham R., Thurrowgood S., Howard D. L., A hidden portrait by Edgar Degas. Sci. Rep. 6, 29594 (2016). 27490856 10.1038/srep29594 PMC4973632

[16] Blažek J., Vlašic O., Zitová B., Improvement of the visibility of concealed features in misregistered NIR reflectograms by deep learning. IOP Conf. Ser. Mater. Sci. Eng. 364, 012058 (2018).

[17] Closer to Van Eyck, http://closertovaneyck.kikirpa.be/ghentaltarpiece/#home [accessed 21 January 2019].

[18] Pizurica A., Platisa L., Ruzic T., Cornelis B., Dooms A., Martens M., Dubois H., Devolder B., De Mey M., Daubechies I., Digital image processing of the Ghent altarpiece: Supporting the painting’s study and conservation treatment. IEEE Signal Process. Mag. 32, 112–122 (2015).

[19] J. Lang, A. Middleton, *Radiography of Cultural Material* (Routledge, 2005).

[20] Padfield J., Saunders D., Cupitt J., Atkinson R., Improvements in the acquisition and processing of x-ray images of paintings. Natl. Gallery Tech. Bull. 23, 62–75 (2002).

[21] R. Yin, D. Dunson, B. Cornelis, B. Brown, N. Ocon, I. Daubechies, Digital cradle removal in X-ray images of art paintings, in *2014 IEEE International Conference on Image Processing (ICIP)* (IEEE, 2014), pp. 4299–4303.

[22] Hyvärinen A., Oja E., Independent component analysis: Algorithms and applications. Neural Netw. 13, 411–430 (2000). 10946390 10.1016/s0893-6080(00)00026-5

[23] Fevotte C., Godsill S. J., A bayesian approach for blind separation of sparse sources. IEEE Trans. Audio Speech Lang. Process. 14, 2174–2188 (2006).

[24] Zibulevsky M., Pearlmutter B. A., Blind source separation by sparse decomposition in a signal dictionary. Neural Comput. 13, 863–882 (2001). 11255573 10.1162/089976601300014385

[25] Bobin J., Starck J.-L., Fadili J., Moudden Y., Sparsity and morphological diversity in blind source separation. IEEE Trans. Image Process. 16, 2662–2674 (2007). 17990743 10.1109/tip.2007.906256

[26] E. J. Candès, X. Li, Y. Ma, J. Wright, Robust principal component analysis? J. ACM 58, 11 (2011).

[27] Grais E. M., Erdogan H., Regularized nonnegative matrix factorization using gaussian mixture priors for supervised single channel source separation. Comput. Speech Lang. 27, 746–762 (2013).

[28] N. Bertin, R. Badeau, G. Richard, Blind signal decompositions for automatic transcription of polyphonic music: NMF and K-SVD on the benchmark. in *Proceedings of International Conference on Acoustics, Speech and Signal Processing*, ICASSP 2007, Honolulu, Hawaii, USA, 15 to 20 April 2007.

[29] P.-S. Huang, M. Kim, M. A. Hasegawa-Johnson, P. S maragdis, Deep learning for monaural speech separation, in *2014 IEEE International Conference on Acoustics, Speech and Signal Processing*, ICASSP, Florence, Italy, 4 to 9 May 2014, pp. 1562–1566.

[30] Deligiannis N., Mota J. F. C., Cornelis B., Rodrigues M. R. D., Daubechies I., Multimodal dictionary learning for image separation with application in art investigation. IEEE Trans. Image Process. 26, 751–764 (2017). 27831873 10.1109/TIP.2016.2623484

[31] Z. Sabetsarvestani, F. Renna, F. Kiraly, M. Rodrigues, Source separation in the presence of side information: Necessary and sufficient conditions for reliable de-mixing, in *2018 IEEE Global Conference on Signal and Information Processing* (GlobalSIP) (2018).

[32] Wang Z., Bovik A. C., Mean squared error: Love it or leave it? A new look at signal fidelity measures. IEEE Signal Process. Mag. 26, 98–117 (2009).

[33] M. Abadi, A. Agarwal, P. Barham, E. Brevdo, Z. Chen, C. Citro, G. S. Corrado, A. Davis, J. Dean, M. Devin, S. Ghemawat, I. Goodfellow, A. Harp, G. Irving, M. Isard, Y. Jia, R. Jozefowicz, L. Kaiser, M. Kudlur, J. Levenberg, D. Mané, R. Monga, S. Moore, D. Murray, C. Olah, M. Schuster, J. Shlens, B. Steiner, I. Sutskever, K. Talwar, P. Tucker, V. Vanhoucke, V. Vasudevan, F. Viégas, O. Vinyals, P. Warden, M. Wattenberg, M. Wicke, Y. Yu, X. Zheng, TensorFlow: Large-scale machine learning on heterogeneous systems (2015); Software available from tensorflow.org.

[34] Wieseman M. E., Rembrandt’s portrait (s?) of Frederik Rihel. Natl. Gallery Tech. Bull. 31, 96–111 (2010).

[35] Dunkerton J., Roy A., Interpretation of the X-ray of du Jardin’s ‘portrait of a young man’. Natl. Gallery Tech. Bull. 6, 19–25 (1982).

[36] J. Dunkerton, M. Spring, Catalogue 8: The death of Actaeon. *National Gallery Technical Bulletin* 36, 104–115 (2015). With contributions from R. Billinge, H. Howard, G. Macaro, R. Morrison, D. Peggie, A. Roy, L. Stevenson and N. von Aderkas.

[37] M. Spring, R. Billinge, L. Treves, N. von Aderkas, C. Higgitt, A. van Loon, J. Dik, Goya’s portraits in the national gallery: Their technique, materials and development. *National Gallery Technical Bulletin* 37, 78–104 (2016). With contributions from R. Billinge, H. Howard, G. Macaro, R. Morrison, D. Peggie, A. Roy, L. Stevenson and N. von Aderkas.

[38] P. Isola, J.-Y. Zhu, T. Zhou, A. A. Efros, Image-to-image translation with conditional adversarial networks, in *2017 IEEE Conference on Computer Vision and Pattern Recognition (CVPR)* (IEEE Computer Society, 2017), pp. 5967–5976.

[39] Pix2pix image-to-image translation, https://ml4a.github.io/guides/Pix2Pix/ [accessed 21 January 2019].

